# No increase in headache after previous intracranial infections: a historical cohort study (The Nord-Trøndelag Health Survey)

**DOI:** 10.1186/1129-2377-14-S1-P148

**Published:** 2013-02-21

**Authors:** M Linde, HA Langnes, K Hagen, K Bergh, LJ Stovner

**Affiliations:** 1 Department of Neuroscience, Norwegian University of Science and Technology, Norway; 2 Department of Microbiology, St. Olavs University Hospital, Trondheim, Norway

## Introduction

Despite the absence of robust scientific evidence, it is today generally accepted that the acute headache typical for intracranial infections can develop into permanent headache complaints.

## Objective

The widespread concept of chronic post-infection headache was explored in the first, large, longitudinal, population-based study.

## Methods

Data on confirmed exposure to intracranial infections amongst all adult inhabitants in a geographical area during a 20-year period were assembled from hospital records. Surviving individuals were later invited to the third Nord-Trøndelag Health Survey (HUNT 3), where 39,690 (42%) of 94,194 invited inhabitants aged ≥20 years responded to a validated headache questionnaire. Using logistic regression, the 1-year prevalence of headache and its subtypes according to the diagnostic criteria of the International Headache Society was assessed and compared between those with and without previous confirmed intracranial infection. Age and sex were used as covariates.

## Results

Overall, 43 participants were identified with earlier intracranial infection, whereof three had more than one infection: bacterial meningitis (n=19), lymphocytic meningitis (n=18), encephalitis (n=9), and brain abscess (n=1).The mean interval from infection to participation in HUNT 3 was 11.2 (range 1.5-19.7) years. There was no significant increase in the prevalence of headache (OR 1.10, 95% CI 0.58-2.07), its subtypes (migraine, or tension-type headache), or chronic daily headache (OR 1.85, 95% CI 0.45-7.68) amongst participants with previous intracranial infection compared with the surrounding population.

## Conclusion

This study challenges the existence of chronic post-bacterial meningitis headache and does not indicate the presence of other long-term headaches induced by intracranial infection.

## Conflict of interest

None.
